# Photoswitchable Spasers with a Plasmonic Core and Photoswitchable Fluorescent Proteins

**DOI:** 10.1038/s41598-019-48335-6

**Published:** 2019-08-27

**Authors:** Walter N. Harrington, Marina V. Novoselova, Daniil N. Bratashov, Boris N. Khlebtsov, Dmitry A. Gorin, Ekaterina I. Galanzha, Vladimir P. Zharov

**Affiliations:** 1 Arkansas Nanomedicine Center, University of Arkansas for Medical Sciences, Little Rock, AR 72205 USA; 2 Skolkovo Institute of Science and Technology, Moscow, Russia; 3 Saratov State University, Saratov, Russia; 4 Institute of Biochemistry and Physiology of Plants and Microorganisms, Saratov, Russia

**Keywords:** Imaging, Optical spectroscopy, Biophotonics

## Abstract

Photoswitchable fluorescent proteins (PFPs) that can change fluorescence color upon excitation have revolutionized many applications of light such as tracking protein movement, super-resolution imaging, identification of circulating cells, and optical data storage. Nevertheless, the relatively weak fluorescence of PFPs limits their applications in biomedical imaging due to strong tissue autofluorecence background. Conversely, plasmonic nanolasers, also called spasers, have demonstrated potential to generate super-bright stimulated emissions even inside single cells. Nevertheless, the development of photoswitchable spasers that can shift their stimulated emission color in response to light is challenging. Here, we introduce the novel concept of spasers using a PFP layer as the active medium surrounding a plasmonic core. The proof of principle was demonstrated by synthesizing a multilayer nanostructure on the surface of a spherical gold core, with a non-absorbing thin polymer shell and the PFP Dendra2 dispersed in the matrix of a biodegradable polymer. We have demonstrated photoswitching of spontaneous and stimulated emission in these spasers below and above the spasing threshold, respectively, at different spectral ranges. The plasmonic core of the spasers serves also as a photothermal (and potentially photoacoustic) contrast agent, allowing for photothermal imaging of the spasers. These results suggest that multimodal photoswitchable spasers could extend the traditional applications of spasers and PFPs in laser spectroscopy, multicolor cytometry, and theranostics with the potential to track, identify, and kill abnormal cells in circulation.

## Introduction

Spasers (surface plasmon amplification by stimulated emission of radiation), with their ability to generate a super-bright, monochromatic stimulated emission in a nanoscale volume, have shown advantages in sensing, imaging, and nanomedicine compared to conventional optical sources^1–9^. Spasers can serve as high contrast probes for integrated diagnostics and therapy (theranostics) of different biological targets, in particular, drug-resistant triple-negative breast cancer cells^5^. Laser-induced photothermal-based transient vapor nanobubbles around overheated absorbing spaser core can amplify photoacoustic (PA) effects and stimulated emission in diagnostic application with simultaneous photomechanical destruction of abnormal cells though damaging of the vital cellular structures during the fast and localized expansion and collapse of nanobubbles. The clinical values of the nanobubbles themselves were recently demonstrated in patients with melanoma through a ~1,000-fold improvement in the circulating tumor cell (CTC) detection limit and the physical destruction of up to 96% of CTCs without harming healthy cells^10^. However, the performance of spasers in traditional laser applications such as high resolution spectroscopy and multicolor microscopy is limited by the slow progress in developing spasers with fast tunable and/or switchable wavelengths. On the other hand, photoswitchable fluorescent proteins (PFPs) that undergo a colour change in response to light, such as Dendra2, PSmOrange, and mEos2, among others, have led to breakthroughs in tracking molecules, organelles, and genetically engineered cells that in turn have significantly enhanced studies of protein dysfunction, cytoskeletal disorders, and neurodegenerative diseases^11–17^. Quick photoswitching of PFPs in fast-moving circulating tumor cells (CTCs) in tumor-bearing animals has allowed the monitoring of real-time dynamics of CTCs released from primary tumors, identification of dormant cells, and imaging of CTCs colonizing a primary tumor (self-seeding) or existing metastasis (reseeding)^15^. However, the low quantum yield efficiency of most PFPs and their limited multifunctionality and quenching effects could restrict their diagnostic applications in areas such as cancer and infection diagnostics due to the strong auto-fluorescent background of blood and tissue^18–20^.

To overcome these limitations, we propose the novel concept of photoswitchable spasers by utilizing PFPs as an active medium structured around a plasmonic core. We verify here the potential of photoswitching spontaneous and stimulated emissions in such nanostructures below and above the spasing threshold, respectively. Strong absorption of the plasmonic core can allow us to use spaser as a photothermal contrast agent in imaging-related application. We anticipate broad biomedical application of the photoswitchable spasers with tunable stimulated emissions and photothermal contrasts similarly to PFPs and lasers in spectroscopy, microscopy, and cytometery, with added advantages such as a smaller size, brighter emission, and wider multifunctionality.

## Results

**Figure 1:**
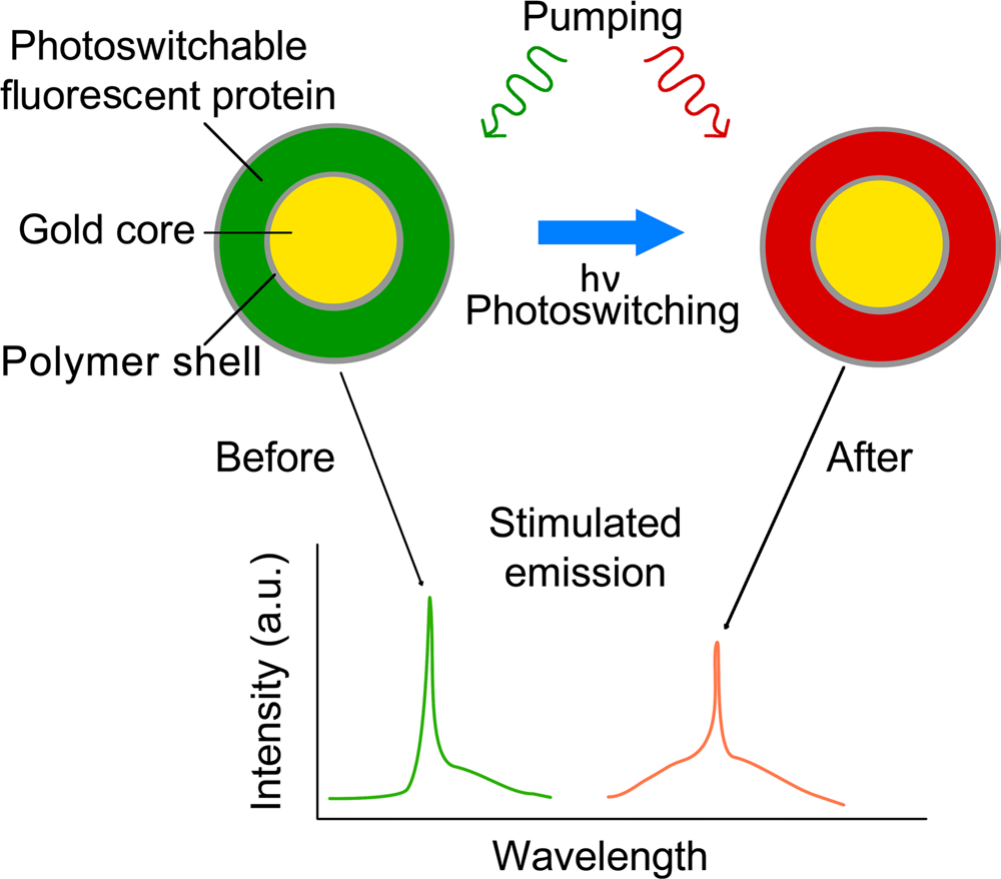
Principle of photoswitchable spasers. Spaser consisting of a plasmonic core with a photoswitchable protein layer (top). Photoswitching of spontaneous and stimulated spaser emission from one PFP state (green fluorescence; left) to another (red fluorescence; right).

The concept of photoswitchable spasers is based on the use of a plasmonic core surrounded by PFPs (Fig. 1). The stimulated emission can first be created using PFPs as an active medium in a non-activated (i.e., not photoswitched) state (Fig. 1, top). After photoswitching the spontaneous emission of the PFP to longer wavelengths (e.g., from green to red), spaser-associated generation of stimulated emission can then be feasible in the shifted spectral range if spasing conditions^1,2^ are met (Fig. 1, bottom). In general, this spaser can generate stimulated emission in the spectral range determined by the overlapping absorption spectra of plasmonic core and active medium emission spectra^2^.

**Figure 2:**
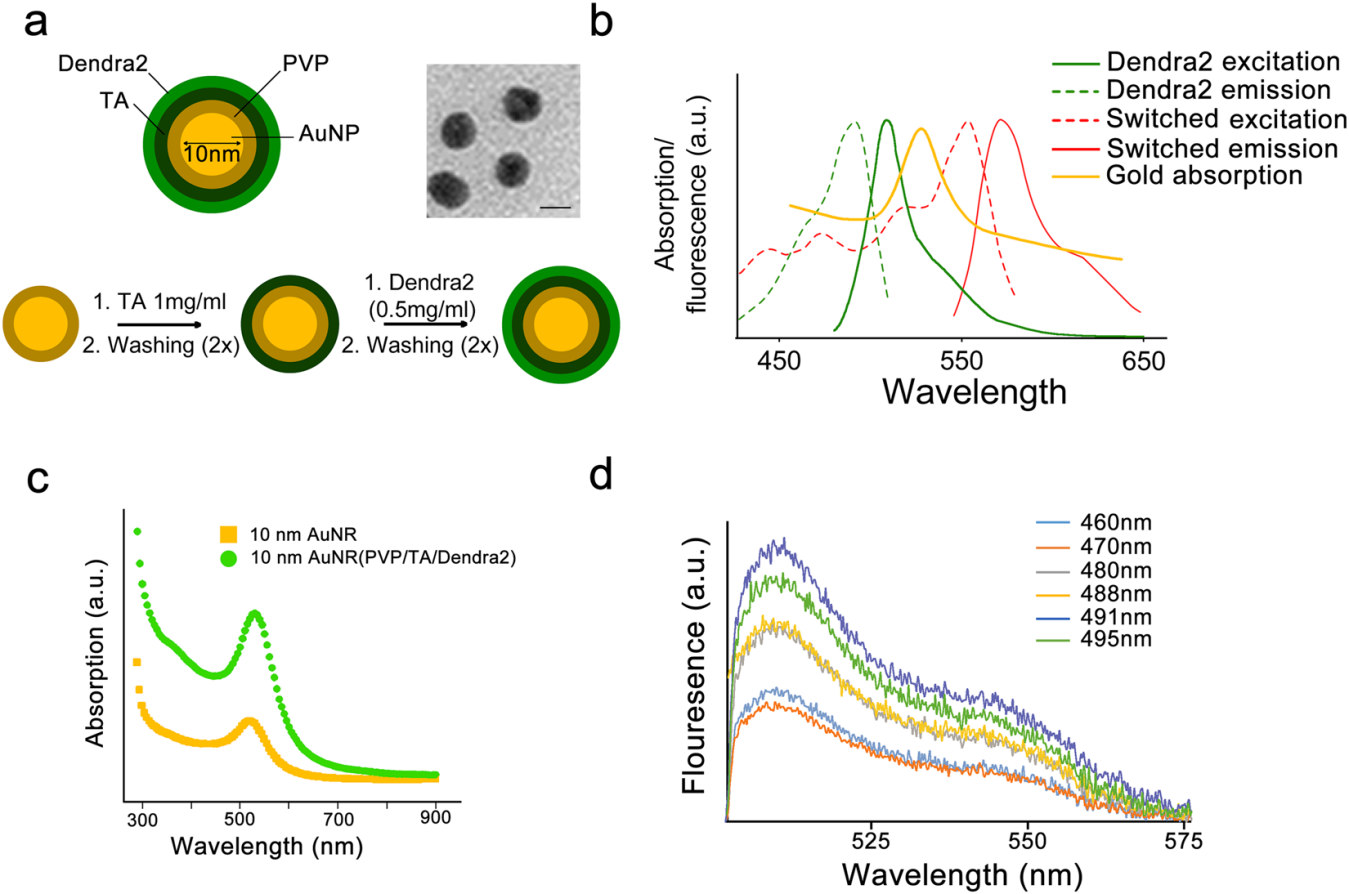
Schematic of photoswitchable spasers. (**a**) Diagram of a spaser consisting of 10-nm gold nanoparticle core with tannic acidstabilized with polyvinylpyrrolidone and doped with Dendra2 (top left). TEM image of individual spasers (top right, scale bar, 10 nm). Illustration of layer-by-layer deposition for core–shell formation (bottom). (**b**) Absorption spectra of gold core (yellow) and absorption (dotted lines) and fluorescence (solid lines) spectra of Dendra2 before (green) and after (red) photoswitching using a continuous wave 405-nm laser. (**c**) Absorption spectra of spaser (green) compared to spectra of gold core (yellow). (**d**) Spontaneous emission (fluorescence) from spasers at different wavelength pulse excitations from an optical parametric oscillator.

The proof of principle (Fig. 1) was demonstrated by synthesizing a multilayer nanostructure on the surface of a spherical gold core with a non-absorbing, nanodimensional polymer shell. The photoswitchable fluorescent protein (PFP) Dendra2 was dispersed in the matrix of a biodegradable polymer. Specifically, we synthesized a spaser consisting of a 10-nm-diameter spherical gold core with polymer layers of tannic acid (TA) stabilized with polyvinylpyrrolidone (PVP) using a well-established procedure termed layer-by-layer (LBL) assembly^20–24^. Our LBL strategy for coating the nanoparticles is illustrated in Fig. 2a.

First, gold nanoparticles (AuNPs) stabilized by PVP were used as the spaser core. The AuNPs were spherical, homogeneous, and free of aggregates. The size of nanoparticles was 10 nm, as determined by dynamic light scattering, which was well within the margin of error for our measurement of 12 nm via transmission electron microscopy (TEM) (Fig. 2, top right).

To link PFPs to AuNPs, a hydrogen-bonded PVP–TA shell was chosen, as TA reacts with PVP^21^ and is able to precipitate proteins by hydrogen bonding and hydrophobic interactions^22–24^. Moreover, TA is a safe, food-grade compound that is used in various biomedical applications, including hemostatic coatings, cell encapsulation, and as nanocontainers for drug delivery systems. TA was deposited on the PVP-coated gold surface to facilitate the binding of the subsequent layers^23^. Thereafter, the nanoparticles were added consecutively to PFP solutions. Finally, PFP was added to complete the final layer of the shell. This layer was doped with a Dendra2 solution at 3.4 mg/mL (Fig. 2a), which has excitation (absorption) and emission spectral bands around the absorption spectra of the spherical AuNPs used as the spaser core (Fig. 2b). The addition of Dendra2 to the gold core did not noticeably change the absorption spectra of the spaser, suggesting that the plasmonic absorption exceeds the absorption of Dendra2 at a similar spectral range (Fig. 2c). Pump excitation of PFPs alone and of spasers with gold cores and PFPs was performed using a tunable optical parametric oscillator (OPO) in a broad spectral range from visible to near-infrared (e.g., from 450 nm to 800 nm)^15^. By changing the pump wavelengths, we optimized the excitation wavelength, demonstrating a maximum excitation intensity near 491 nm (Fig. 2d).

To test the photoswitching capacity of spasers, we used both a conventional arc lamp with a filter and a continuous wave (CW) laser diode at a wavelength of 405 nm. In particular, we have determined that fast photoswitching at a time scale of a few milliseconds can be achieved at a laser power of 11 mW without photo-bleaching, due to the short exposure time, which is in line with our previous study^15^.

**Figure 3:**
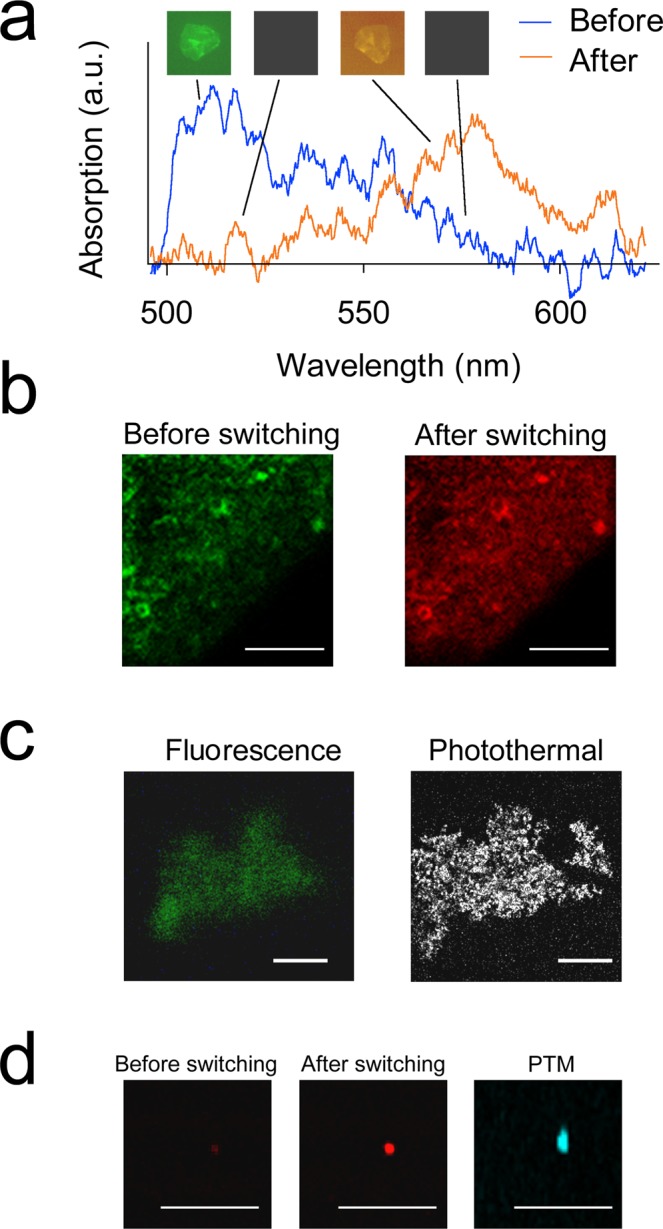
Photoswitching of spontaneous emissions from spasers. (**a**) Emission spectra of spaser cluster before (green) and after (orange) photoswitching with a 405-nm laser. Insets show fluorescent images of spaser clusters. (**b**) Fluorescence of individual spasers in the green (left) or red (right) spectral range before and after photoswitching, respectively. (**c**) Fluorescent images of individual spasers in the green spectral range (left) and photothermal images of the same spasers (right). (**d**) Fluorescence of non-photoswitched (left) and photoswitched (middle) spaser clusters in red spectral range and photothermal microscopy (PTM) image of the same spaser cluster (right). Scale bars, 10 μm.

Photoswitching of Dendra2 below the spasing threshold of 37 ± 6 mJ/cm^2^ led to spectral shifting of spaser stimulated emission that coincided with its conventional fluorescent spectra (Fig. 3a), with a maximum intensity at 511 nm and 576 nm (Fig. 2b). Due to the relatively weak emission of the first samples caused by a low Dendra2 concentration, we used a spaser microcluster for fluorescent imaging (Fig. 3a, insets). Optimizing the synthesis procedure with a higher PFP concentration allowed us to obtain images of individual spasers with the fluorescence technique in the green spectral range (Fig. 3b,c, left) and of spaser clusters in the red spectral range (Fig. 3d, middle). Using advanced two-beam (pump–probe) PT microscopy based on the non-radiative relaxation of absorbed energy into heat^5,15^, we confirmed that spasers can serve as high-contrast PT agents (Figs 3c, right; 4d, right) due to strong light absorption by the plasmonic core.

**Figure 4:**
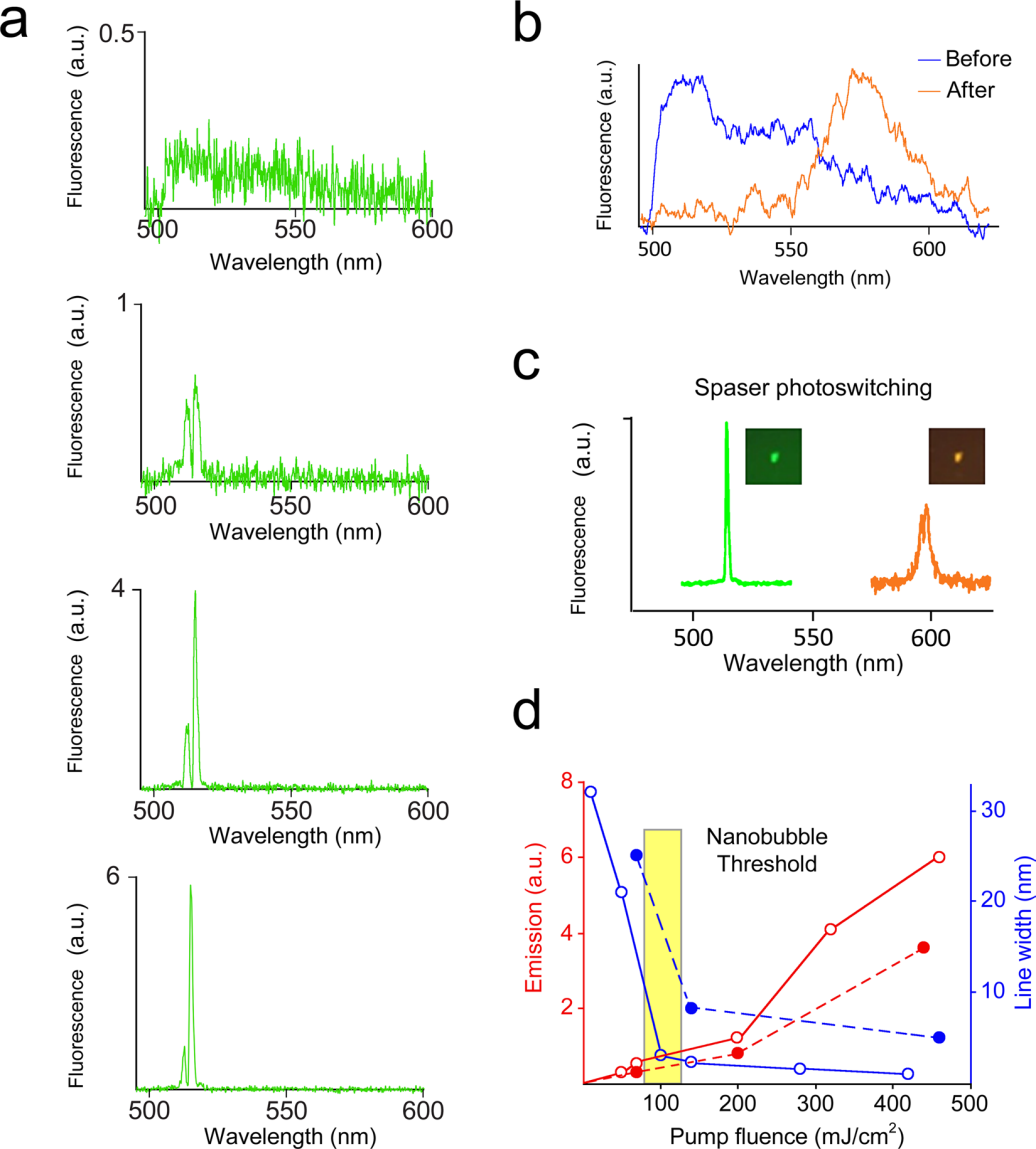
Photoswitching of stimulated emissions from spasers. (**a**) Emission spectra of non-photoswitched spasers below the spasing threshold (30 mJ/cm^2^, top) and above the threshold, with pump energy fluence of approximately up to 500 mJ/cm^2^ (from second to fourth panel) using a single pump pulse at 491 nm. (**b**) Spontaneous emission spectra of spasers before (blue) and after (orange) photoswitching at a pump energy fluence of 70 mJ/cm^2^ (i.e., below threshold) at a wavelength of 491 nm and 30 pulse averaging. (**c**) Stimulated emission spectra of spasers using pump laser at a wavelength of 491 nm and 540 nm before (green) and after (red) photoswitching using UV light and energy fluence of 190 mJ/cm^2^, and 240 mJ/cm^2^, respectively. (**d**) Stimulated emission from spaser suspension. Red: input-output (light out – pump [L-P]) curve of spasing. Blue: emission linewidth of spasing. Empty circles connected by solid lines and solid circles connected by dashed lines indicate data before and after photoswitching, respectively. Standard deviations (SD) for intensity and linewidth are in the range of 18–26%.

Increasing the pump energy above the spasing threshold led to the appearance of ultra-sharp emission spectra for non-photoswitched spasers. The pump energy provided increased intensity for these peaks and narrowing of their linewidth from 30 nm, with the presence of 2 to 3 overlapping peaks (Fig. 4a, green), to 1 nm, with 1 dominant peak (Fig. 4c, red). Above the threshold, the L (light)-P (power emission) demonstrated a straight line, typical for spasers (Fig. 4d, red)^1,2^. Further increasing the pump energy led to the previously described formation of vapor nanobubbles around spasers, indicated by specific imaging patterns and nonlinear photothermal signal enhancement^5,16,25,26^. The appearance of nanobubbles was accompanied by nonlinear enhancement of stimulated emission intensity and dramatic linewidth narrowing (Fig. 4d). Using procedures similar to those described above, we observed that it was possible to photoswitch the spontaneous emission below the spasing threshold (Fig. 4b, green and red), and above the threshold, it was possible to photoswitch the stimulated emission from a green to red state using 10 ns pulse pump OPO at 491 nm and 540 nm, respectively (Fig. 4c). The dependence of stimulated emission on the pumping parameters demonstrated linear and non-linear^4^ nanobubble-associated behavior and a linewidth narrowing from 35 nm to 1 nm and 3.5 nm at wavelengths of 515 nm and 597 nm, respectively (Fig. 4d), also typical for spasers^1,2,5^.

## Discussion

Despite the development of many advanced spasers, most of the research in this area still focuses on exploring new schematics^2–9^ rather than new applications^4,5^. Recently, we demonstrated the first biological applications of spasers with a fixed wavelength (i.e., with one color) that consisted of plasmonic nanoparticle cores as the optical resonator, with a silica or polymer layer doped with a low-toxicity active-gain medium such as uranine and indocyanine green (ICG), which are both approved for use in humans^5,7^. Here, we introduce photoswitchable spasers that can further extend the applications of spasers, with a focus on their multicolor capacity. Aside from the photoswitching of spaser stimulated emissions, we also simultaneously demonstrated the ability to image them with photothermal imaging, due to the strong plasmonic absorption of their cores (Figs 3c, right; 3d, right). Thus, the phenomenon of heat loss that was previously considered a limitation of spasers^9^ is shown here to be a positive characteristic of photoswitchable spasers. Previously, we introduced the concept of photothermally switchable plasmonic nanoparticles with weak fluorescence and controllable spectral shifts in absorption in response to light using ultrasharp photothermal resonances^5,15,25,26^. We believe that the combination of fluorescence, photoswitching of PFPs, and absorption in the plasmonic core can enhance both modalities for using integrated fluorescence, photothermal, and potentially photoacoustic microscopy and cytometry^15,16^ (basic physical principles of both methods are similar)^25,26^. The photoswitchable spasers, as small as 20 to 30 nm, can be excited by a single nanosecond laser pulse in both states (non-photoswitched and photoswitched) within a short timeframe (a few nanoseconds)^2,5^. The relatively high pumping level above the threshold in our current study can be explained by the small spaser size (30–60 nm), relatively low initial PFP emission intensity below the lasing threshold, and a low concentration of PFPs in the silica layer. We are currently working on further improving these parameters. Further developments may also involve the use different PFPs (e.g., PSmOrange or mEos2) and plasmonic core shapes (e.g., gold nanorods or nanoshells) that absorb in the near-infrared range and can be tunable by changing the nanoparticle size and shape^5,9,25,26^

## Methods

### Materials

Tannic acid (TA), polyvinylpyrrolidone (PVP, mW~10000), bovine serum albumin (BSA), silver nitrate (AgNO3, 99.99%), tetrachloroauric acid (HAuCl4, >99%), sodium borohydrate (96%), phosphate buffer saline (PBS), and sodium chloride were purchased from Sigma-Aldrich. Isoascorbic acid (IAA, >99%) was purchased from Fluka. All chemicals were used as received without further purification. Deionized (DI) water (specific resistivity higher than 18.2 MΩcm) from a Milli-Q plus 185 (Millipore) water purification system was used to make all solutions.

### Spaser synthesis

A layer-by-layer assembly method was used to prepare the nanostructures, as previously described in the literature^20–23^. AuNPs with an average diameter of 10 nm were used as the spaser core. The nanoparticles were produced with the Frens method^27^. Briefly, 4 mL of 1% sodium citrate solution was added to 100 mL of boiling 0.01% HAuCl_4_ solution. For nanoparticle stabilization, 1 mL 0.1% PVP solution was added to the prepared AuNPs. The spaser structure (Fig. 2a) was obtained by adsorption of 1 mL TA (2 mg/mL in H_2_O) and then Dendra2 (0.5 mg/mL) onto the surface of the nanoparticle core, stabilized with PVP. After each of the adsorption steps, the suspension was centrifuged and washed twice with pure water. For the supernatants, the equation [protein (mg/ml) = 1.55 A_280_ − 0.76 A_260_] was used to determine protein concentration. The PFP Dendra2 was obtained as a purified protein product from Creative BioMart at a concentration of 3.4 mg/mL and was used to prepare a solution in phosphate-buffered saline (PBS).

### Fluorescence measurements

To study the spontaneous and stimulated emission, samples (e.g., spaser solution) were loaded on microscope slides with 120-µm and 1-µm path lengths. The samples were irradiated by OPO (below) at different wavelengths and spots of different diameters from 1 µm to 100 µm. For spectroscopic measurements, we used a fiber-optic-based spectrometer (OceanOptics USB4000) with optical resolution of Δλ ~ 0.1 nm (FWHM) or AvaSpec-2048 TEC-FT-2 [Δλ ~ 0.7 nm (FWHM)]. Fluorescent images of nanoparticles were obtained with a Leica TCS SP8 X inverted confocal microscope (Leica Microsystems). An HC PL APO 100x/1.44 oil-immersion objective was used.

### Experimental PTM setup

Spaser characterization was performed with photothermal microscopy (PTM), as described previously^16,25^. Briefly, the PTM module was built on the technical platform of an Olympus IX81 inverted microscope (Olympus America, Inc.) containing a tunable optical parametric oscillator (OPO; Opolette HR 355 LD, Opotek, Inc.) with the following parameters: 420–2,200-nm tunable spectral range; 5-ns pulse width; 100-Hz pulse repetition rate; and a 0.1–10^3^ mJ/cm^2^ fluence range. Laser beams were focused into the sample with various magnification objectives, including a 100× oil-immersion objective (DPlan 100, NA 1.25, Olympus, Inc.) and 40× objective (Ach, NA 0.65, Olympus, Inc.). Pump (OPO) laser-induced temperature-dependent variations in the refractive index caused a collinear He–Ne laser probe beam (model 117 A, Spectra-Physics, Inc.; wavelength 633 nm; power 1.4 mW) to defocus, reducing the intensity of the beam at its center. The probe laser light was collected after it was exposed to the sample using either a 100× water-immersion objective (LUMPlanFl 100, NA 1.00, Olympus, Inc.) or a 40× objective (Ph3 DL, NA 0.55, Nikon Inc) and was detected by a photodiode (PDA36A, Thorlabs, Inc) with a 50-µm pinhole. Typical PTM-related signals from the photodiode in linear mode are characterized by an initial peak associated with rapid heating of nanoparticles and with a slower exponential tail corresponding to the cooling of the target. In nonlinear mode, laser-induced nanobubbles around overheating nanoparticles lead to the appearance of sharp negative peaks associated with the refraction and scattering of the probe beam on the nanobubbles^25,26^. The photothermal images are constructed by acquiring the signals from a sample as it undergoes scanning in *x* and *y* dimensions using a two-dimensional stage (H117 ProScan II, Prior Scientific, Inc.) with a scanning step of 0.25–1 µm. The signals were recorded using a 200-MHz analog-to-digital converter board (PCI-5152, National Instruments Corp.) and analyzed with custom software (based on LabVIEW 8.5, National Instruments Corp.). A Dell Precision 690 workstation provided signal acquisition and processing, synchronization of the excitation laser, and translation-stage control. Resolution was determined by the microscope objective itself (e.g., ~0.7 µm at 20×, NA 0.4; and ~250 nm at 100×, NA 1.25).

### Spectral measurements

Extinction spectra were measured by UV-visible spectrometry with the Synergy H1 Multi-Mode Reader (BioTek Instruments, Inc., USA). All measurements were performed using disposable 96-well plates at 24 °C. For spectroscopic measurements, we used a fiber-optic-based spectrometer (OceanOptics USB4000) with an optical resolution of Δλ ~ 0.1 nm (FWHM) or an AvaSpec-2048 TEC-FT-2 (Δλ ~ 0.7 nm [FWHM]).

### Transmission electron microscopy

Transmission electron microscopy images were obtained with a Libra-120 transmission electron microscope (Carl Zeiss, Germany) at 120 kV at the “Simbioz” CCU at the Institute of Biochemistry and Physiology of Plants and Microorganisms, Russian Academy of Sciences. ImageJ software was used to measure the diameters of nanoparticles.

### Statistical analysis

Results are expressed as the mean ± standard deviation (SD), and the the confidence interval was determined with at least three independent experiments (P_0.25_ 0.95). Statistica 5.11 (StatSoft, Inc.), MATLAB 7.0.1 (MathWorks), and LabVIEW (National Instruments) were used for the statistical calculations. In the figures, we indicate only the average SD in the caption to simplify data presentation.
